# Methylphenidate Treatment Decreases Borderline Personality Symptoms in Adolescents: Evidence Beyond ADHD Symptom Improvement

**DOI:** 10.1192/j.eurpsy.2026.10197

**Published:** 2026-07-31

**Authors:** R. E. Ildız, Ö. F. Akça, C. Sharp

**Affiliations:** 1 Child and Adolescent Psychiatry, Erzincan Mengücek Gazi Training and Research Hospital, Erzincan; 2 Child and Adolescent Psychiatry, Necmettin Erbakan University, Konya, Türkiye; 3 Psychology, University of Houston, Houston, United States

## Abstract

**Introduction:**

Attention-deficit/hyperactivity disorder (ADHD) and borderline personality disorder (BPD) are frequently comorbid and share overlapping clinical features. While psychotherapy remains the primary treatment for BPD, there is no established pharmacological intervention. Methylphenidate and atomoxetine are effective in managing ADHD symptoms.

**Objectives:**

This study aimed to evaluate the impact of methylphenidate and atomoxetine on borderline personality traits in adolescents diagnosed with ADHD.

**Methods:**

Adolescents with ADHD who were initiated on methylphenidate or atomoxetine were followed for 12 weeks. Prior to and following treatment, participants completed the Borderline Personality Features Scale for Children (BPFS-C), the Difficulties in Emotion Regulation Scale (DERS), the Barratt Impulsiveness Scale–Short Form (BIS-S), and the Adolescent Dissociative Experiences Scale (ADES). Parents completed the Turgay DSM-IV–Based Disruptive Behavior Disorders Rating Scale (T-DSM-IV-S). Due to the small number of participants receiving atomoxetine (n = 5), only data from those treated with methylphenidate (n = 45) were included in the final analyses.

**Results:**

Post-treatment assessments revealed significant reductions in BPFS-C subscales and total scores, as well as in the total scores of DERS, BIS-S, and ADES. Improvements were more pronounced in female participants, particularly in the BPFS-C self-harm subscale, the total BPFS-C score, and BIS-S scores. After controlling for ADHD symptom change and sex, significant reductions remained in the BPFS-C emotional dysregulation subscale, total BPFS-C score, and total scores of DERS, BIS-S, and ADES.

**Conclusions:**

Methylphenidate treatment led to significant reductions in borderline personality traits among adolescents with ADHD. These improvements were independent of ADHD symptom reduction and were especially evident in female participants.

**Disclosure of Interest:**

None Declared

